# *Alu* insertion polymorphisms shared by *Papio* baboons and *Theropithecus gelada* reveal an intertwined common ancestry

**DOI:** 10.1186/s13100-019-0187-y

**Published:** 2019-11-26

**Authors:** Jerilyn A. Walker, Vallmer E. Jordan, Jessica M. Storer, Cody J. Steely, Paulina Gonzalez-Quiroga, Thomas O. Beckstrom, Lydia C. Rewerts, Corey P. St. Romain, Catherine E. Rockwell, Jeffrey Rogers, Clifford J. Jolly, Miriam K. Konkel, The Baboon Genome Analysis Consortium, Jeffrey Rogers, R. Alan Harris, Muthuswamy Raveendran, Yue Liu, Shwetha Murali, Tauras P. Vilgalys, Jerilyn A. Walker, Miriam K. Konkel, Vallmer E. Jordan, Cody J. Steely, Thomas O. Beckstrom, Gregg W. C. Thomas, Kymberleigh A. Pagel, Vikas Pejaver, Claudia R. Catacchio, Nicoletta Archidiacono, Mario Ventura, Alessia Marra-Campanale, Antonio Palazzo, Oronzo Capozzi, Archana Raja, John Huddleston, Veronica Searles Quick, Anis Karimpour-Fard, Dominik Schrempf, Marc de Manuel Montero, Konstantinos Billis, Fergal J. Martin, Matthieu Muffato, Georgios Athanasiadis, Christina Bergey, Andrew Burrell, Jade Cheng, Laura Cox, James Else, Yi Han, Gisela H. Kopp, Maximilian Kothe, Kalle Leppälä, Angela Noll, Jera Pecotte, Lenore Pipes, Karen Rice, Christopher E. Mason, Todd Disotell, Jane Phillips-Conroy, Lutz Walter, Kasper Munch, Thomas Mailund, Mikkel Schierup, Carolin Kosiol, Tomas Vinar, James M. Sikela, Dietmar Zinner, Christian Roos, Clifford J. Jolly, Predrag Radivojac, Roscoe Stanyon, Mariano Rocchi, Evan E. Eichler, Bronwen Aken, Matthew W. Hahn, Mark A. Batzer, Tomas Marques-Bonet, Jenny Tung, Donna M. Muzny, Richard A. Gibbs, Kim C. Worley, Mark A. Batzer

**Affiliations:** 1 Department of Biological Sciences, Louisiana State University, 202 Life Sciences Building, Baton Rouge, Louisiana, 70803 USA; 2 Human Genome Sequencing Center, Baylor College of Medicine, Houston, TX 77030 USA; 3 Department of Molecular and Human Genetics, Baylor College of Medicine, Houston, TX 77030 USA; 4 Department of Anthropology, New York University, New York, NY 10003 USA; 5 Department of Genetics & Biochemistry, Clemson Center for Human Genetics, Clemson, SC 29634 USA

**Keywords:** Retrotransposon, Evolutionary biology, Primate phylogeny, *Alu* element

## Abstract

**Background:**

Baboons (genus *Papio*) and geladas (*Theropithecus gelada*) are now generally recognized as close phylogenetic relatives, though morphologically quite distinct and generally classified in separate genera. Primate specific *Alu* retrotransposons are well-established genomic markers for the study of phylogenetic and population genetic relationships. We previously reported a computational reconstruction of *Papio* phylogeny using large-scale whole genome sequence (WGS) analysis of *Alu* insertion polymorphisms. Recently, high coverage WGS was generated for *Theropithecus gelada.* The objective of this study was to apply the high-throughput “poly-Detect” method to computationally determine the number of *Alu* insertion polymorphisms shared by *T. gelada* and *Papio*, and vice versa, by each individual *Papio* species and *T. gelada*. Secondly, we performed locus-specific polymerase chain reaction (PCR) assays on a diverse DNA panel to complement the computational data.

**Results:**

We identified 27,700 *Alu* insertions from *T. gelada* WGS that were also present among six *Papio* species, with nearly half (12,956) remaining unfixed among 12 *Papio* individuals. Similarly, each of the six *Papio* species had species-indicative *Alu* insertions that were also present in *T. gelada*. In general, *P. kindae* shared more insertion polymorphisms with *T. gelada* than did any of the other five *Papio* species. PCR-based genotype data provided additional support for the computational findings.

**Conclusions:**

Our discovery that several thousand *Alu* insertion polymorphisms are shared by *T. gelada* and *Papio* baboons suggests a much more permeable reproductive barrier between the two genera then previously suspected. Their intertwined evolution likely involves a long history of admixture, gene flow and incomplete lineage sorting.

## Background

The phylogenetic position of the gelada (*Theropithecus gelada*) has been debated since the species was first scientifically described in 1835 by Rüppell. Originally named *Macacus gelada,* it was later placed in a genus of its own by I. Geoffroy Saint-Hilaire (1843) [1] where it remains today as the only extant species of *Theropithecus* [2]. By contrast, there are currently six recognized species of *Papio* baboons distributed across most of sub-Saharan Africa [3–5]. Evidence from morphological comparisons and mitochondrial and whole genome sequencing (WGS) all support a primary phylogenetic division into northern (*P. anubis*, *P. papio* and *P. hamadryas*) and southern (*P. ursinus*, *P. cynocephalus* and *P. kindae*) clades [5–7]. The genetics of the baboon species complex have been studied much more extensively [4–14] than that of the mountain dwelling geladas of the Ethiopian highlands [15–20]. *Theropithecus* is estimated to have diverged from a *Papio-*like ancestor about 4–5 million years ago (mya) based on fossil evidence [2, 4, 21, 22] and analysis of mitochondrial DNA [23], while extant *Papio* species began to diversify about 2 mya [5, 7, 9, 24].

A complex history of evolution has been reported among extant species within the genus *Papio* [5–7, 25–28], in which mitochondrial and phenotypically-based phylogenies of the six currently recognized extant species frequently conflict. Recently, the Baboon Genome Analysis Consortium published a study of the complex population history of *Papio* baboons based on whole genome sequences, providing evidence for multiple episodes of introgression and admixture throughout radiation of the genus and a long history of genetic exchange among diverging lineages that were presumably phenotypically distinct [6].

Primate specific *Alu* retrotransposons are well-established genomic markers for the study of population genetic and phylogenetic relationships [27, 29–40]. *Alu* element insertions are considered unique events, have a known directionality where the ancestral state is known to be the absence of the element, and are relatively inexpensive to genotype [33, 41–45]. *Alu* insertions shared by individuals or species are widely accepted as largely being inherited from a common ancestor. The amplification of *Alu* elements has been ongoing in primate genomes since the origin of the Order, about 65 mya [42, 46, 47]. *Alu* elements mobilize via a “copy and paste” mechanism through an RNA intermediate, a process termed “target-primed reverse transcription” (TPRT) [48]. We recently reported a computational reconstruction of *Papio* phylogeny using 187,000 *Alu* insertions identified through a large-scale whole genome sequence analysis [26]. This study not only determined the most likely branching order within *Papio* with high statistical support, but also quantified the number of *Alu* insertions supporting alternative topologies, demonstrating the efficacy of whole genome computational analysis of *Alu* polymorphisms to identify and investigate complexities in phylogenetic relationships.

During the early stages of the Baboon Genome Analysis Consortium [6] an analysis of the [Panu_2.0] genome of *Papio anubis* revealed an occasional *Alu* element insertion that appeared to be present in *T. gelada* DNA based on PCR, while also remaining polymorphic among the six *Papio* species. Although intriguing given the estimated 4–5 mya divergence between the two genera, with no other WGS data available at the time for further computational screening, these insertions were set aside as being uninformative for resolving phylogenetic relationships within *Papio*. Recently, we have generated high coverage WGS data for an individual *Theropithecus gelada* (Sample name 36168, BioProject PRJNA251424, submitted by Baylor College of Medicine). Therefore, the objective of this study was to apply the “polyDetect” method [26] to computationally determine the number of *Alu* insertion polymorphisms shared by the representative *T. gelada* genome and 12 individuals representing the genus *Papio*. Our approach targeted recently integrated *Alu* insertions present in *T. gelada* yet polymorphic within *Papio* and absent from rhesus macaque [Mmul8.0.1]. *Alu* insertions recent enough to remain polymorphic among *Papio* species would be expected to have integrated after the split from *Theropithecus* and therefore be absent from *Theropithecus*. Similarly, *Theropithecus*, with a much smaller effective population size [20], would be expected to have its own set of lineage-specific insertions. Observations of a large number of *Alu* insertions present in both genera that remain unfixed in all species would suggest a long history of ancient admixture, extensive incomplete lineage sorting, or on-going hybridization [44]. Here, we have computationally ascertained a dataset of *Alu* insertions present in the *Theropithecus gelada* WGS data that also remained polymorphic among 12 *Papio* baboons representing all six species. This analysis prompted a reciprocal computational comparison of WGS of each *Papio* individual to determine the number of *Alu* insertion polymorphisms shared exclusively between each *Papio* species and *T. gelada*.

Locus specific PCR analyses were performed on a DNA panel which included samples from all six *Papio* species, *T. gelada* and rhesus macaque (*Macaca mulatta*) as an outgroup to provide experimental support for the computational findings.

## Methods

### WGS samples

Whole-genome sequencing was performed by the Baylor College of Medicine Human Genome Sequencing Center. All samples were sequenced to an average coverage of 32.4x and minimum of 26.3x [6]. The same dataset described in Jordan et al. (2018) [26] for 12 *Papio* individuals was used in this analysis along with WGS from a representative *T. gelada* genome. These samples are listed in Additional file 1. We used two individuals from each of the six extant *Papio* species (we randomly selected two individuals from *P. anubis* and *P. kindae*) to conduct our computational analysis; along with WGS data from the rhesus macaque sample used to build the recent *M. mulatta* assembly [Mmul8.0.1] and WGS data for one *Theropithecus gelada* (isolate 891096; sample name 38168; adult female captive born at the Bronx Zoo; NCBI BioProject PRJNA251424; Accession: SAMN06167567). WGS data were accessed from the NCBI-SRA database as described previously [26].

### Computational *Alu* detection

We used the “polyDetect” computational pipeline [26] to perform our analysis. Our approach targeted recently integrated *Alu* insertions present in *T. gelada* yet polymorphic within *Papio* and absent from rhesus macaque [Mmul8.0.1]. The approximate chromosomal position of each candidate insertion was estimated using a split-read method as described previously [26]. Briefly, for the alignment phase, we used BWA-MEM version 0.7.17-r1188 [49] to map the sequencing reads to a consensus *Alu*Y sequence obtained from Repbase [50]. The *Alu* portion of each candidate split-read was cleaved allowing the remaining unique flanking sequence to be aligned to the rhesus macaque genome assembly [Mmul8.0.1] using bowtie2 version2.3.2 [51]. Split-reads were categorized as sequences that mapped uniquely to the *Alu*Y consensus sequence and the [Mmul8.0.1] assembly. The resulting genotypes, generated for all individuals in our panel, isolated thousands of phylogenetically informative markers. Data for these loci were sorted by the number of *Alu* insertions common to *T. gelada* and any two to twelve *Papio* individuals. For purposes of the present analyses, those present in all 12 *Papio* individuals were considered fixed present in the dataset and eliminated from this portion of the study. For the reciprocal comparison, the *Alu* insertions detected in both individuals of a single *Papio* species, as reported previously in Jordan et al. (2018) [26], were sorted by their [Mmul8.0.1] predicted insertion coordinates and cross-referenced with coordinates from the *T. gelada* WGS reads to identify candidate shared insertion polymorphisms. These are listed in Additional file 1, Worksheet “*Papio-Theropithecus*.”

### Statistical analysis of *Alu* insertion polymorphisms

*Alu* insertions predicted to be shared by *T. gelada* and any two to eleven of the twelve *Papio* individuals were considered polymorphic in the genus *Papio* and retained for further analysis. To determine if any particular species or clade had significantly different numbers of shared insertions with *T. gelada*, we performed a one-way analysis of variance (ANOVA) in Excel (alpha set at 0.05). A separate ANOVA was performed for each of the ten data bins representing two to eleven individuals. ANOVA “groups” were defined as either six *Papio* species with two individuals each, or two *Papio* clades (North / South) with six individuals each. If a significant ‘between group’ difference was detected, we followed with a Bonferroni post-hoc test in Excel, selecting the “t-Test: Two-sample assuming equal variances” function to perform a two-tailed t-test for *P* ≤ 0.05. All *P* values were recorded in Additional file 1: Table S1.

### Candidate *Alu* element selection and oligonucleotide primer design

We randomly selected 150 candidate *Alu* insertion polymorphisms from the first comparison (A: ascertained from the *T. gelada* WGS and polymorphic among *Papio* baboons) for in-house oligonucleotide primer design as described previously [52]. From the second comparison (B: present in WGS of both individuals of a single *Papio* species and shared in *T. gelada*) we randomly selected about 10% of the candidate loci identified from each of the six *Papio* species, but no less than five loci from each species, for primer design. Oligonucleotide primers for PCR were designed using the predicted insertion coordinates from the rhesus macaque genome [Mmul8.0.1] since that was the “reference” genome used to map the *T. gelada* and *Papio* WGS reads. Suitable primer pairs were then analyzed against the *Papio anubis* baboon genome [Panu_2.0] using the “In-Silico PCR” tool in BLAT [53] through the University of California Santa Cruz (UCSC) Genome Browser [54]. If no PCR product was identified due to mismatches in the primer sequence, the primer pairs were analyzed by In-Silico PCR using the [Mmul8.0.1] assembly to obtain the predicted PCR product. This entire amplicon sequence was then analyzed using BLAT against the *P. anubis* genome [Panu_2.0] and checked for mismatches in order to design alternative oligonucleotide primers to help ensure PCR amplification in *Papio* baboons. Using this method we obtained estimates for our expected PCR product sizes in [Mmul8.0.1] and [Panu_2.0] (Additional file 2). Oligonucleotide primers for PCR were obtained from Sigma Aldrich (Woodlands, TX).

### Polymerase chain reaction assays

The primate DNA panel used for PCR analyses was comprised of three *P. anubis*, one *P. hamadryas,* two *P. papio,* two *P. cynocephalus,* two *P. ursinus*, two *P. kindae*, one *T. gelada*, and a *Macaca mulatta*. A human (HeLa) sample was used as a positive control and TLE (10 mM Tris / 0.1 mM EDTA) was used as a negative control. Information about the samples is provided in Additional file 2 including their common name, origin, and ID.

A total of 172 *Alu* insertion polymorphisms were retained in the dataset for PCR analyses. We used a subset of the computationally-derived *Alu* insertion polymorphisms ascertained from either A) *T. gelada* WGS and predicted to be shared in *Papio*, (*N* = 96); or B) *Papio* species WGS and predicted to be shared in *T. gelada,* (*N* = 52). We also included *N* = 24 *Alu* loci previously ascertained from the reference genome of *Papio anubis* [Panu_2.0] (12 loci each from [6, 52]) in which PCR results indicated the *Alu* insertion was present in *T. gelada* while remaining polymorphic among the six *Papio* species.

Oligonucleotide primers for PCR were designed using Primer3 software, either manually [55] for most of the Panu_2.0 derived candidate loci or using a modified version [56]. PCR amplifications were performed in 25 μl reactions containing 25 ng of template DNA; 200 nM of each oligonucleotide primer; 1.5 mM MgCl_2_, 10x PCR buffer (1x:50 mM KCl; 10 mM TrisHCl, pH 8.4); 0.2 mM dNTPs; and 1–2 U *Taq* DNA polymerase. PCR reactions were performed under the following conditions: initial denaturation at 94 °C for 60 s, followed by 32 cycles of denaturation at 94 °C for 30 s, 30 s at annealing temperature (57 °C – 61 °C), and extension at 72 °C for 30 s. PCRs were completed with a final extension at 72 °C for 2 min. Twenty microliter of each PCR product were fractionated by size in a horizontal gel chamber on a 2% agarose gel containing 0.2 μg/ml ethidium bromide for 60 min at 185 V. UV-fluorescence was used to visualize the DNA fragments and images were saved using a BioRad ChemiDoc XRS imaging system (Hercules, CA). Following gel electrophoresis, genotypes were recorded in an Excel spreadsheet as (1, 1) for homozygous present, (0, 0) for homozygous absent, or (1, 0) for heterozygous. “Missing data” was coded as (− 9, − 9). Genotypes for these 172 loci are shown in Additional file 2; Worksheet “Genotypes.”

### Validation of computational predictions

Our DNA panel for locus-specific PCR analyses did not include samples from every WGS individual analyzed. Because our representative *T. gelada* individual differed from that supplying the WGS sample used for *Alu* ascertainment, we used genotype data from PCR analyses for ten *Papio* individuals on our DNA panel to estimate the validation rate of the computational predictions (Additional file 3). Based on these results, we implemented an additional filtering step on the data in an attempt to minimize the number of false predictions, while continuing to ensure that our interpretation of the computational results was correct. This filter involved re-analyzing the read files for the dataset of *Alu* insertions present in *T. gelada* WGS and imposed a minimum length requirement of 30 bp of unique 5′ flanking sequence adjacent to the predicted *Alu* insertion for the call to be retained. These post-filtered data were sorted as before for the number of shared *Alu* insertions between *T. gelada* and any two to twelve *Papio* individuals. The set of candidate loci determined to be present in both individuals of a single *Papio* species (as reported previously in Jordan et al. 2018), that were also computationally predicted to be shared with *T. gelada,* were also subjected to the filtering step and those retained were then screened against the [Panu_2.0] baboon genome to eliminate those shared in the *P. anubis* reference genome.

### *Alu* subfamily analysis

*Papio* lineage-specific *Alu* subfamilies evolved from older *Alu*Y subfamilies after the baboon stem lineage diverged from its common ancestor with the rhesus macaque [52]. Identification of *Alu* subfamilies and the corresponding sequence divergence can provide insight regarding the approximate age of an *Alu* insertion event [52, 57]. This study included 24 loci ascertained from the baboon genome assembly [Panu_2.0] and another 16 ascertained from the *T. gelada* WGS with complete *Alu* sequence available. PCR data indicated that 15 of the 24 [Panu_2.0] set and 8 of the 16 WGS set met the study criteria of being polymorphic among *Papio* baboons and shared by *T. gelada*. These 23 polymorphic loci were analyzed for *Alu* subfamily affiliation. Using the genome coordinates in BED format we uploaded a custom track to the UCSC Genome Browser [54] using the Table Browser function. The complete *Alu* sequence was obtained in FASTA format. Subfamily identification for these elements was determined using an in-house RepeatMasker library [58] (http://www.repeatmasker.org; last accessed November 2019) developed in Steely et al. (2018) [52].

## Results

### Computational *Alu* detection

**Fig. 1:**
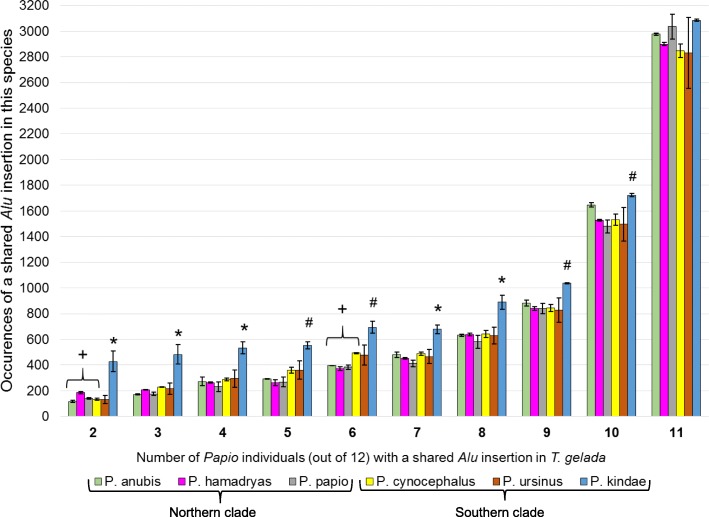
The number of times a *T. gelada*-ascertained *Alu* insertion polymorphism was predicted to be shared in a *Papio* species when shared in any of 2 to 11 of the 12 *Papio* individuals. Vertical bars are the average of the two individuals of a given species +/− the standard deviation (error bars). No *Papio* individuals are preferentially excluded from having shared insertions with *T. gelada* in any category. In bin two, *P. hamadryas* has significantly more shared insertions than *P. anubis*, *P. papio*, and *P. cynocephalus* (+: *P* ≤ 0.05). In bin six, *P. cynocephalus* has significantly more shared insertions than the three northern species, *P. anubis*, *P. hamadryas* and *P. papio* (+: *P* ≤ 0.05). Across bins 2 to 10 shared insertions are predicted in *P. kindae* significantly more often than all other five *Papio* species (*) or all except *P. ursinus* (#) (*P* ≤ 0.05)

Our split-read methods predicted 27,700 *Alu* insertions in *T. gelada* WGS data shared among the 12 *Papio* individuals but absent from rhesus macaque [Mmul8.0.1] (Additional file 4). Because our objective was to target recently integrated *Alu* insertions present in *T. gelada* yet polymorphic within *Papio*, we eliminated 14,744 (53%) that were present in all twelve *Papio* individuals. We retained the remaining 12,956 shared by any of two to eleven of the twelve *Papio* individuals for further analysis. To determine if any particular *Papio* species or clade was favored or excluded for shared insertion events with *T. gelada*, we sorted the raw output for the number of shared *Alu* elements in each bin of 2 to 11 individuals (Table 1). Then we counted the number of times a shared insertion was predicted in each *Papio* individual (Table 1). For example, when an *Alu* insertion was predicted to be present in any 5 of the 12 *Papio* individuals and absent from the other 7, we found 294 instances where one of the five individuals with the insertion was *P. anubis* LIV5. All 12 *Papio* individuals shared hundreds of *Alu* insertion polymorphisms with *T. gelada* in all categories. The average of the two individuals of each species + / - the standard deviation is plotted in Fig. 1. A one-way ANOVA with Bonferroni correction detected significant between-group differences for test bins 2 to 10, but not for bin 11. In bin 2, *P. hamadryas* has more shared insertions with *T. gelada* than do *P. anubis, P. papio*, or *P. cynocephalus*, while in bin 6, *P. cynocephalus* has more shared insertions than the three northern species (Fig. 1; Additional file 1: Table S1). As a group, the northern and southern clades appear to have similar representation overall except as detected in bins 5 and 6 (of 12) in which the southern clade has significantly more shared insertions, on average, than the northern clade (*P* ≤ 0.05; Additional file 1: Table S1). However, the most consistent statistical finding across all bins was for the two *P. kindae* individuals. *P. kindae* has significantly more shared *Alu* insertions with *T. gelada* than all other five *Papio* species in bins 2 to 4 and 7 to 8, while significantly more in all except *P. ursinus* in the remaining bins 5, 6, 9 and 10 (Fig. 1; Additional file 1: Table S1).

**Table 1:** Number of *T. gelada Alu* insertion polymorphisms shared in *Papio* individuals

A.	B.	Northern clade						Southern clade					
		*P. anubis*		*P. hamadryas*		*P. papio*		*P. cynocephalus*		*P. ursinus*		*P. kindae*	
		LIV5	L142	97124	97074	28547	30388	16066	16098	28697	28755	34449	34474
2	1139	112	122	192	179	136	146	127	140	155	111	486	372
3	989	174	169	210	205	166	184	227	231	249	185	537	430
4	944	296	248	268	261	206	259	282	297	343	247	567	502
5	839	294	290	248	280	241	294	342	375	413	310	574	534
6	938	396	396	360	381	370	396	491	497	531	421	727	662
7	851	495	466	448	456	395	430	497	480	505	428	702	655
8	991	626	638	631	645	546	617	623	663	677	584	849	929
9	1171	899	865	830	851	811	869	824	862	894	759	1040	1035
10	1881	1659	1635	1531	1522	1442	1516	1501	1563	1590	1405	1732	1714
11	3213	2980	2971	2890	2907	2966	3104	2811	2884	3025	2636	3079	3090
	**12,956**												

The number of *Alu* insertion polymorphisms ascertained from *T. gelada* and not fixed in all 12 *Papio* individuals was calculated to be 12,956. The distribution of these when shared between any of 2 to 11 of the 12 *Papio* individuals (column A, 2 to 11) is shown in column B. The sum of the values in column B is 12,956. The ID for each *Papio* individual is shown at the top of the twelve adjacent columns, for each of the six *Papio* species, separated by northern and southern clades. The numbers in each column represent the number of times that the shared insertion with *T. gelada* was predicted in that individual. For example, when an *Alu* insertion was predicted to be shared in 4 of the 12 individuals and absent from the other 8, one of the four (column A, row 4) was *P. anubis* LIV5 296 times and one of the four was *P. kindae* 34474 (BZ11050) 502 times. All 12 *Papio* individuals share hundreds of *Alu* insertion polymorphisms with *T. gelada* in all categories. No *Papio* individuals are preferentially excluded from having shared insertions with *T. gelada*. ANOVA detected between-group differences in bins 2–10, but not bin 11. *P. kindae* has significantly more shared insertion events with *T. gelada* than all other five *Papio* species in bins 2 to 4 and 7 to 8, while significantly more in all except *P. ursinus* in the remaining bins 5, 6, 9 and 10. See Fig. 1

These findings prompted us to perform the reciprocal database comparison (B) between the *Papio* WGS *Alu* analyses reported in Jordan et al. (2018) [26] and the current WGS *Alu* database for *T. gelada*. In that study, *P. kindae* was found to have the most ‘species-indicative’ *Alu* insertions with 12,891 elements identified in both *P. kindae* individuals and absent from both the two individuals of all the other five *Papio* species. We cross-referenced those 12,891 *P. kindae Alu* loci with the database of 27,700 *T. gelada Alu* loci to determine if any were shared exclusively between *P. kindae* and *T. gelada* and identified 236 (1.83%) cases. We performed the same cross-reference analyses for the other five *Papio* species and found that each of the six *Papio* species had *Alu* insertions shared exclusively with *T. gelada*. *P. kindae* had significantly more shared insertions than the other five *Papio* species (*P* < 0.05) (Table 2). The predicted insertion coordinates and sample IDs are listed in Additional file 1, Worksheet “*Papio-Theropithecus*.”

**Table 2:** Number of *Papio* species-indicative *Alu* insertion polymorphisms shared with *Theropithecus gelada*

*Papio* species	Number of species-indicative *Alu* insertions	Number shared in *T. gelada*	%	Z-score	one-tailed *P*-value
*P. anubis*	4645	34	0.73%	−0.6821	0.2476
*P. hamadryas*	8060	101	1.25%	0.1802	0.4285
*P. papio*	10,873	68	0.63%	−0.2445	0.4036
*P. cynocephalus*	2794	26	0.93%	−0.7851	0.2162
*P. ursinus*	9545	57	0.60%	−0.3861	0.3498
*P. kindae*	12,891	236	1.83%	1.9176	0.0276 *

The number of *Alu* insertion polymorphisms shared in WGS of *Theropithecus gelada* that were reported [26] to be exclusive to one *Papio* species and absent from the other five. *P. kindae* has significantly more such shared elements with *T. gelada* than the other five *Papio* species (*P < 0.05). Z-scores are calculated based on the mean, 87, and standard deviation equal to 77

### Candidate loci and PCR analyses

A subset of 150 *T. gelada* computationally-derived candidate *Alu* insertion events were selected for PCR analyses. The oligonucleotide primer design pipeline selected suitable primer pairs using the [Mmul_8.0.1] genome as the mapped reference. After screening these primer pairs against the baboon genome assembly [Panu_2.0], a total of 105 loci were analyzed by PCR for *Alu* presence / absence within *Papio* and *T. gelada*, with 96 generating interpretable results (Additional file 2). PCR based genotypes revealed that 60 of these 96 loci (62%) met the objective criteria of being polymorphic for insertion presence / absence among *Papio* baboons and also being shared in a representative *T. gelada* individual, KB10538 from the San Diego Zoo (DNA was not available for WGS individual 38168). Allele frequency calculations on these 60 loci showed that *P. hamadryas* sample 97124 and *P. kindae* sample 34474 (BZ11050) had the highest counts of shared insertions with 25 and 24%, respectively, while the average across the other *Papio* samples was 18% (Additional file 2, Worksheet “allele frequency”). Given that these loci were randomly selected from thousands of candidates, the fact that PCR shows *P. kindae* to have one of the highest frequencies of alleles shared with *T. gelada* supports the computational predictions reported in Table 1.

**Fig. 2:**
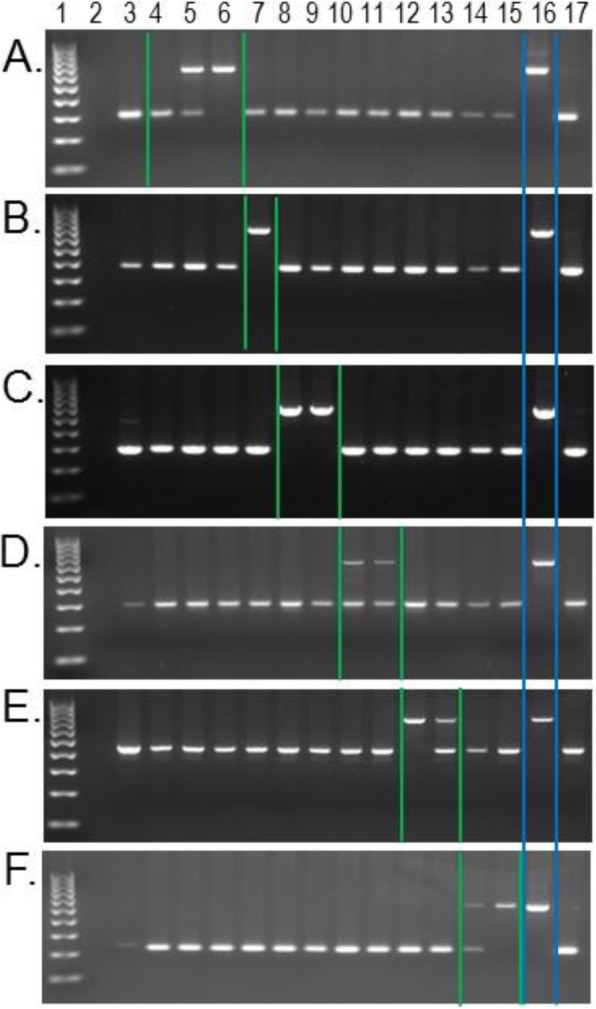
*Papio* species-indicative *Alu* insertion polymorphisms shared in *Theropithecus gelada*. Lanes: 1- 100 bp ladder, 2- TLE (negative control), 3- Human (HeLa), 4- *P. anubis* (27861 Panu_2.0 reference individual), 5- *P. anubis* (L142), 6- *P. anubis* (LIV5), 7- *P. hamadryas* (97124), 8- *P. papio* (28547), 9- *P. papio* (30388), 10- *P. cynocephalus* (16066), 11- *P. cynocephalus* (16098), 12- *P. ursinus* (28697), 13- *P. ursinus* (28755), 14- *P. kindae* (34474; BZ11050), 15- *P. kindae* (34472; BZ11047), 16- *T. gelada* (KB10538), 17- *Macaca mulatta*. **a** olive baboon locus AnuGel_12; **b** hamadryas locus HamGel_76; **c** Guinea baboon locus PapioGel_38; **d** Yellow baboon locus YelGel_11; **e** chacma baboon locus ChacmaGel_43; **f** kinda baboon locus KindaGel_199. Green bars outline the *Papio* species with the *Alu* present (upper band); the blue bar outlines the *Alu* present band in *T. gelada*

The second subset of PCR candidates was selected from the dataset of *Papio* species-indicative elements shared with *T. gelada* (Table 2). Because we did not have DNA samples from every WGS sample analyzed, including the *T. gelada*, we randomly selected approximately 10% of the candidate loci from each *Papio* species for PCR analysis, with a minimum of five per species. A total of 52 loci from this dataset were analyzed by PCR with 49 generating interpretable results (Additional file 2). PCR results confirmed 26 of these loci contained the candidate *Alu* insertion in the predicted *Papio* species and the representative *T. gelada* individual KB10538 (Additional files 2 and 3). Although 26 of 49 is only about a 53% confirmation rate from within the candidate loci selected, they provide clear evidence that this particular phenomenon of shared *Alu* insertion polymorphisms exists in nature, and that each *Papio* species has multiple *Alu* insertions also shared in *T. gelada* but not yet observed in the other five *Papio* species. An example of this scenario for each of the six *Papio* species is illustrated with an agarose gel image in Fig. 2.

In addition to the candidate *Alu* insertion polymorphisms computationally ascertained in this study, subsets A and B, we also retained 24 loci from previously published studies (12 loci each from [6, 52]) that were ascertained from the olive baboon genome [Panu_2.0] in which PCR experiments indicated the *Alu* insertion might be shared by *Papio* and *Theropithecus*. PCR results using the current DNA panel confirmed that 15 of these 24 met the objective criteria of being polymorphic for insertion presence / absence among *Papio* baboons while also being shared in our representative *T. gelada* sample. All 172 loci in this study (96 + 52 + 24) were confirmed by PCR to be absent in rhesus macaque.

### Validation of computational predictions

We analyzed genotype data for the *Papio* individuals on our DNA panel to determine the validation rate of the computational predictions (Additional file 3). Of the 96 loci in this dataset that were ascertained from WGS of *T. gelada*, a total of 206 instances of a filled allele being shared with a *Papio* individual on our DNA panel were predicted computationally. No PCR amplification occurred in 3 cases, leaving 203 predicted shared cases to analyze. 145 (71%) of the 203 were confirmed by PCR while 58 (29%) of the 203 were shown by PCR to be false predictions. Of the 58 false predictions, nearly three-quarters (*N* = 43) occurred within 22 loci in which all individuals genotyped as absent for the insertion. A review of all the read files, split-reads and paired-end reads used to make these predictions, provided some clues as to why some predictions were validated by PCR while others were not. PCR-validated predictions typically had multiple supporting reads with at least 40–50 bp of unique 5′ flanking sequence adjacent to the head of the *Alu* insertion. Predictions not confirmed by PCR tended to have very short (≤ 25 bp) 5′ flanking sequence. This suggests that a lack of flanking sequence to accurately map the split-reads to unique sequence is the likely cause for the majority of the false predictions. Seven (7.3%) of the 96 loci were considered ‘false negative’ in that they were not computationally detected in all 12 *Papio* individuals (considered polymorphic), but the PCR results indicated the insertion was present in all the *Papio* individuals on the DNA panel (Additional file 3). This type of error is likely caused by a lack of supporting reads for those individuals such that the insertion is simply not detected, rather than being “predicted absent” by the polyDetect method.

To determine the role of 5′ flanking sequence length on the number of false predictions, we re-analyzed the dataset of 27,700 *Alu* insertions present in *T. gelada* WGS that were computationally predicted to be present in any of two to twelve *Papio* individuals and absent from rhesus macaque [Mmul8.0.1]. We implemented a ‘read filter’ requiring a minimum of 30 bp of 5′ flanking sequence adjacent to the predicted *Alu* (See Methods). These post-filtered data were sorted as before for the number of *Alu* insertions shared by *T. gelada* and any two to twelve *Papio* individuals. The post-filtered equivalent of Table 1 is available in Additional file 1: Table S2 and the associated *P* value for each bin is shown on the same worksheet as Additional file 1: Table S3. The post-filtered equivalent of Fig. 1, using data from Additional file 1: Table S2, is shown in Additional file 1: Figure S1. The number of acceptable candidate loci dropped from 27,700 to 22,875, with 10,422 (45.6%) of those determined to be present in all 12 *Papio* individuals and the remaining 12,453 (54.4%) were determined to be polymorphic among any two to eleven *Papio* individuals. Although the number of elements in any particular bin shifted somewhat with gains or losses due to the filter requirement, the overall results and interpretation of those results remained the same. All 12 *Papio* individuals share dozens of *Alu* insertion polymorphisms with *T. gelada*. Also, as with the original analyses, *P. kindae* still has significantly more shared *Alu* insertions with *T. gelada* than any of the other five *Papio* species in most bins while significantly more in all except *P. ursinus* in bins 4–6. The observable consequences of the filtering step appear to be a reduction in the number of acceptable reads for *P. anubis* sample L142, as compared to the other *Papio* individuals. Also, the mean values of shared insertions with *T. gelada* now favor the southern clade over the northern clade more consistently (bins 3–7) than in the previous analyses (bins 5–6). Of the 22 loci containing 43 of the 58 false predictions in the previous analyses, 16 loci and 34 of the 43 false calls were omitted by the filtering step. The number of false predictions was reduced from 58 to 22 and the false prediction rate dropped from 29 to 11% (Additional file 3). Only one previously validated call was erroneously filtered out. Therefore, the filtered results improved the overall validation rates within this study.

However, the effect of the 30 bp flanking requirement on data reported in Table 2 was more informative. The filter reduced the number of acceptable calls in *P. anubis* sample L142, thus reducing the number found in both *P. anubis* individuals, LIV5 and L142. The consequence was that some loci were eliminated that had already been PCR validated (i.e. Anu-12 and Anu-6; Additional file 3). Alternatively, the number of predicted *P. hamadryas* indicative elements included 7 new loci that were not in the original set because they had previous calls in L142 or other *Papio* individuals that now had been filtered out. Therefore, not only were some reads eliminated, as expected, but this in turn erroneously added loci to each “*Papio*-indicative” category due to previously called reads in other *Papio* individuals that were no longer acceptable under the filter conditions. To obtain a value for each *Papio* species with “high confidence” following the filtering step, we retained only those post-filtered loci also present in the original analyses reported in Table 2, that were also not present in the Panu_2.0 genome. (Additional file 1: Table S4). As before, *P. kindae* still has significantly more shared *Alu* insertions with *T. gelada* than do the other five *Papio* species (*P < 0.05).

In our attempt to minimize the number of false predictions and improve the validation rate of the polyDetect output in this study, we also inadvertently increased the number of ‘false negative’ calls dramatically. That is, the absence of a call (no detection in a WGS individual) does not necessarily mean the “predicted absence” of the *Alu* insertion, only a lack of acceptable mapped reads. Therefore, the filtered results were far less accurate for this metric of the study compared to the first analysis. Also, the errors induced by the filter were more problematic to the overall results of the study than the relatively minor impact of the initial false prediction rate. This highlights the importance of validating methods for data filtering and downstream data processing, and its potential impact on data interpretation. In this case, having a large dataset with overwhelming numbers meant that the overall interpretation was robust to the identified issues.

### *Papio Alu* subfamily distribution

Of the 172 elements PCR-analyzed in this study, only 23 were suitable for *Alu* subfamily analysis. They had the complete *Alu* sequence available from the [Panu_2.0] reference genome and met the study criteria of being polymorphic for insertion presence / absence among *Papio* baboons while also being shared in *T. gelada*. These sequences were analyzed for *Papio Alu* subfamily assignment using an in-house RepeatMasker [58] library developed by Steely et al. (2018) [52]. The RepeatMasker output is available in Additional file 2, Worksheet “RM output”. Most of these subfamilies are generally older ancestral subfamilies as shown by their location near the central nodes of the clusters reported in Steely et al. (2018) [52]. The percent divergence from the respective consensus sequences ranged from 0.3 to 3.9% with the average being 1.8% (≤ 2% divergence is considered relatively young) [59, 60]. Of the 23 loci analyzed, 7 were assigned directly to subfamily *Alu*MacYa3, the central node of cluster 1 matching subfamily 0 [52] and the ancestral node originally discovered in *Macaca mulatta*. Another 11 loci were assigned to *Papio Alu* subfamilies that derived from *Alu*MacYa3. One locus derived from *Alu*Y (3.2% divergence) while the remaining four loci represented different subfamily clusters but were generally from older rhesus macaque subfamilies such as *Alu*YRa4 (Additional file 2).

## Discussion

The close evolutionary relationship between savanna baboons, genus *Papio*, and geladas, genus *Theropithecus*, is well documented [2, 5] although recognized as separate genera based on numerous differences in morphology, social behavior and ecology [4, 16, 19, 21]. Our finding that about half (47–54%) of *Alu* insertions ascertained from a representative *T. gelada* genome have not reached fixation in the *Papio* species is unexpected given a *Theropithecus / Papio* divergence time dating back to 4–5 mya. We also find that each of the six *Papio* species possesses several species-indicative *Alu* insertions (present in both individuals of that species while absent from all ten individuals from the other five species) that are shared inter-generically with *T. gelada*. This implies a long history of incomplete lineage sorting, admixture and gene flow.

During most of the Plio-Pleistocene, *Theropithecus* was present throughout much of non-rainforest Africa. Three subgenera are currently recognized: *T. (Theropithecus*), *T.* (*Simopthecus*), and *T. (Omopithecus)*. Of these, *T.* (*Theropithecus*), including only the extant *T. gelada*, is unknown as a fossil, and may have always been restricted to the Ethiopian highlands. *T. (Omopithecus*) includes only a single recognized species, *T*. *brumpti*, confined to the Early Pleistocene of East Africa. The third subgenus, *T*. (*Simopithecus*), including *T. oswaldi* and closely related species, is extensively distributed in time and space, from ~ 4 mya to ~ 100 kya, and from southern Africa to Algeria, extending into southern Europe and western Asia [2, 4, 22, 61, 62]. Late populations of *T*. (*S.) oswaldi* were probably too large in body mass to breed successfully with *Papio* baboons, but for most of its history, *T*. (*Simopithecus*) was comparable in mass to extant baboons.

Some observations of extant baboons and geladas suggest that even after 4 mya of separate evolution, the possibility of gene flow between them is not completely excluded by an intrinsic barrier. A suspected hybrid individual has been observed in a natural gelada-olive baboon overlap zone [63]. In a zoo environment, completely viable first-generation hamadryas baboon x gelada hybrids of both sexes are reliably reported. While the hybrid males are suspected to be infertile, female hybrids have produced viable offspring by backcrossing to *Papio hamadryas* [64]. Especially during the earlier phases of their long period of co-existence, *Papio x Theropithecus* matings (including with *T. oswaldi*) may have allowed ongoing, low-frequency genetic exchange. Our *Alu* insertion polymorphism data support this hypothesis.

In this study, we also report that *P. kindae* baboons share more *Alu* insertions with *T. gelada* than do the other *Papio* baboons. The reason for this is not well understood and may require further study. Each of the 12 *Papio* genomes was sequenced to an average read depth of 32.4x coverage with minimum coverage 26.3x [6] and therefore it is unlikely that this finding can be attributed to differences in sequence coverage. An *Alu*-based phylogeny of *Papio* species placed *P. cynocephalus,* not *P. kindae,* as most basal within the southern clade [26]. The modern ranges of *P. kindae* and *T. gelada* are geographically far apart [5, 7]. If they adjoined or overlapped, it might suggest recent hybridization between the two taxa. Moreover, all of the *Papio* individuals investigated had dozens of shared insertions with *T. gelada*, including multiple species-indicative loci. None were preferentially excluded. This suggests that modern geography and habitat are not contributing factors to this finding. Using whole genome comparisons within *Papio*, the *P. kindae* genome was found to harbor more species-indicative *Alu* insertions than the other five species and also found to share more *Alu* insertions with members of the northern clade that were absent from the other southern clade members [26]. The history of *P. kindae* is reportedly quite unique among baboons. As part of the Baboon Genome Analysis Consortium [6], the best fitting model using coalescent hidden Markov methods indicated that the history of *P. kindae* includes an ancient admixture event involving a lineage related to extant *P. ursinus* from the southern clade (52% contribution to extant *P. kindae*), with the remaining 48% contribution to extant *P. kindae* originating from an ancient lineage, possibly extinct, belonging to the northern clade [6]. However, other scenarios may also be possible. If extant *P. kindae* is the (now geographically restricted) descendent of a geographically widespread ancestral population that exchanged genes with ancestral populations in the *Theropithecus* lineage and also gave rise to small spin-off populations that expanded one to the north and another to the south, this might also be consistent with the *Alu* evidence presented in this study.

Our analyses of *Alu* subfamily distribution are also consistent with a complex evolutionary history for *Papio*. The ancestral lineages of Asiatic and African papionin monkeys diverged about 8 mya [23]. *Alu* subfamilies rooted with rhesus macaque, meaning that these subfamilies were active prior to the divergence of *Theropithecus* / *Papio* from *Macaca*, such as *Alu*MacYa3, were shown in this study to have recently integrated progeny elements in *Theropithecus* / *Papio*. Many of the 23 *Alu* insertion polymorphisms analyzed for subfamily assignment had < 2% divergence from their respective consensus sequences, providing support for their recent integration. The observation that generally older *Alu* subfamilies have produced the majority of the relatively recent integration events is consistent with the overall estimated divergence timeframe of 4–5 mya. Low *Alu* sequence variation combined with ongoing persistent levels of insertion polymorphism suggest that the *Alu* retrotransposition rate among these lineages has been relatively uniform over a long period of time, possibly driven by a lack of reproductive isolation [65].

This study suggests that *Papio* baboons and *Theropithecus* have a long history of intertwined evolutionary ancestry that likely includes episodes of intergeneric introgression. A precedent for this among other African primates is available by examining the complex origins of the kipunji, *Rungwecebus kipunji*. The kipunji is a papionin primate discovered in Tanzania in 2003. It was initially assigned to the genus *Lophocebus* (arboreal mangabey) based on general morphology and arboreal behavior [66] but genetic studies based on mtDNA from a single specimen from Mount Rungwe indicated the new species was more closely related to baboons, genus *Papio* [67, 68]. The arboreal mangabey-like phenotype of the kipunji combined with a mtDNA profile similar to a yellow baboon, suggested that *Rungwecebus kipunji* originated from a hybridization event between a female yellow baboon (*Papio cynocephalus*) and a *Lophocebus* male mangabey [69]. It was not until genetic material became available from a kipunji individual from the Ndundulu population about 350 km away that new evidence suggested that the two kipunji populations likely have different evolutionary histories [70]. The Ndundulu haplotype is considered to be the ancestral or “true” mitochondrial haplotype while the Mount Rungwe population has experienced more recent and perhaps persistent localized introgression from *Papio*, introducing the observed *Papio* mtDNA haplotype [71]. The example of the kipunji provides a biological precedent with regard to intergeneric introgression among African primates, similar to our findings between genus *Papio* and genus *Theropithecus*.

Following Groves (2001) [3], the tribe Papionini includes macaques (*Macaca*), mandrills (*Mandrillus*), terrestrial mangabeys (*Cercocebus*) and the Highland mangabeys (*Rungwecebus kipunji*) along with three closely related genera *Papio*, *Theropithecus* and *Lophocebus* [23]. Phylogenetic studies of Papionini have generally separated the genera into two clades, one with *Macaca* basal to sister taxa *Cercocebus* and *Mandrillus* and a second clade consisting of *Theropithecus, Papio* and *Lophocebus*, subgenus Papionina [23, 72]*.* Phylogenetic relationships among the three Papionina genera remain unresolved [23, 73]. Some studies have placed *Theropithecus* basal to a clade consisting of *Papio* and *Lophocebus* [73, 74], while other analyses have placed *Theropithecus* and *Papio* as sister taxa, with *Lophocebus* diverging first [23]. The fact that extensive molecular evidence has yet to resolve this phylogeny suggest possible admixture, reticulation and short internode intervals that facilitate incomplete lineage sorting, and possibly inter-generic hybridization among the lineages.

The increasing availability of vast amounts of WGS data has led to many recent studies being conducted based exclusively on computational analyses, without wet-bench experimental validation to support the genomic comparisons [75, 76]. Although these reports are informative, this study demonstrates the need to interpret such results with caution. It is important to keep in mind that although “figures don’t lie”, all forms of data filtering and downstream processing have consequences, some of which are obvious while others are more obscure. Computational data alone may produce interpretable results, but the biological significance of such interpretation should be anchored with experimental evidence when possible. This is especially important when investigating complex phylogenies with an extensive history of admixture and hybridization. Even high quality WGS data from limited sample sizes may not necessarily be representative of the species or genus as a whole, thus molecular validation and adequate sampling are required to support the findings. It is undeniable, however, that the burgeoning availability of WGS data allows greater resolution of complex phylogenies while also recognizing and addressing the impact of confounding factors.

## Conclusions

In this study, we computationally identified over twelve thousand *Alu* insertions polymorphic in *Theropithecus* and *Papio*. Even after incorporating our initial 71% validation rate and possible 7.3% false negative error rate, at least 8500 *Alu* insertions have not reached fixation among the two genera. PCR sequencing based on a small subset of these insertions confirmed over one hundred such cases in support of the computational findings. We also computationally identified over 500 *Papio* species-indicative *Alu* insertions polymorphisms (present in WGS of both individuals of one *Papio* species while being absent from two samples from each of the other five species) that were determined to be shared in *T. gelada*. PCR evidence confirmed numerous cases of this unexpected phenomenon. All six *Papio* species have many *Alu* insertion polymorphisms shared with *T. gelada*, while *P. kindae* has the largest number. This study suggests that *Papio* baboons and *Theropithecus* have a long history of intertwined evolutionary ancestry that likely includes episodes of intergeneric introgression.

## Supplementary information

**Additional file 1.** An Excel file containing different worksheets for the WGS sample list, Tables S1 – S4, Figure S1 and “Papio-Theropithecus”. (XLSX 173kb)

**Additional file 2.** An Excel file with worksheets for DNA samples, oligonucleotide PCR primers, genomic coordinates, genotype data for the PCR experiments, allele frequency and RepeatMasker output. (XLSX 89kb)

**Additional file 3.** An Excel file summarizing the PCR validation of computational predictions. (XLSX 45.6kb)

**Additional file 4.** An Excel file with a list of the 27,700 T. gelada / Papio shared Alu insertions. (XLSX 1.7MB)

## Data Availability

The algorithms used in this study are available on GitHub (https://github.com/papioPhlo/polyDetect). The Additional Information files are available on the online version of this paper and through the Batzer Lab website under publications, https://biosci-batzerlab.biology.lsu.edu/. Additional file 1 is an Excel file containing a WGS sample list, Additional file 1: Tables S1-S4, Additional file 1: Figure S1 and worksheet “*Papio-Theropithecus.”* Additional file 2 is an Excel file with worksheets for DNA samples, oligonucleotide PCR primers, genomic coordinates, genotype data for the PCR experiments, allele frequency and RepeatMasker output. Additional file 3 is an Excel file summarizing the PCR validation of computational predictions. Additional file 4 is an Excel file with a list of the 27,700 *T. gelada / Papio* shared *Alu* insertions.

## Abbreviations

bp: Base pairs
kya: thousand years ago
mya: million years ago
PCR: Polymerase chain reaction
TPRT: Target primed reverse transcription
WGS: Whole genome sequence
